# Using a co-design approach to develop aquatic reactive balance training for fall prevention

**DOI:** 10.1371/journal.pone.0345435

**Published:** 2026-07-15

**Authors:** Anna Ogonowska-Slodownik, Julia O. Faria, Shanuga Thavarajah, Karina Pacholczyk, Birgit Blain, Myra Wiener, Jane Walker, Avril Mansfield, Soo Chan Carusone

**Affiliations:** 1 Faculty of Rehabilitation, Jozef Pilsudski University of Physical Education in Warsaw, Warsaw, Poland; 2 KITE Research Institute, Toronto Rehabilitation Institute – University Health Network, Toronto, Ontario, Canada; 3 Ribeirão Preto Medical School, University of São Paulo, Ribeirão Preto, Brazil; 4 Department of Laboratory Medicine and Pathobiology, University of Toronto, Toronto, Ontario, Canada; 5 Older adult partners, Toronto, Ontario, Canada; 6 Department of Physical Therapy, University of Toronto, Toronto, Ontario, Canada; 7 McMaster Collaborative for Health and Aging, McMaster University, Hamilton, Canada; UFPE: Universidade Federal de Pernambuco, BRAZIL

## Abstract

**Background:**

Falls are a major health concern among older adults, leading to injury, reduced independence, and increased healthcare cost. Reactive balance training can reduce fall risk, but barriers such as fear, joint discomfort, and harness burden limit its use – barriers that training in aquatic environment  may help overcome.

**Objective:**

To design an aquatic reactive balance training (AquaReBal) program for older adults, integrating end-user perspectives to enhance safety, accessibility, and engagement.

**Methods:**

Using a participatory design approach grounded in Participatory Action Research principles, we engaged three older adult partners, physiotherapists, and researchers in iterative phases including literature review, stakeholder consultations, practical pool sessions, and feedback meetings. Data collected through online meetings, surveys, field notes, and observations were reviewed using an inductive qualitative content analysis approach to inform iterative refinement of the intervention, following the Guidance for Reporting Involvement of Patients and the Public 2 framework.

**Results:**

Three older adult partners and a multidisciplinary team co-designed the AquaReBal protocol through two participatory design sessions, one practical pool session, and two internal team sessions. Key recommendations from partners included using a vest instead of a hip belt for perturbations, addressing pool depth visibility, and creating an introductory package with practical information for participants. Partners emphasized safety, instructor support, and social engagement as critical for adherence and satisfaction.

**Conclusion:**

The co-design process enabled the development of an AquaReBal protocol tailored to older adults’ needs and preferences, providing initial insights relevant to future feasibility and implementation.

## Introduction

Falls are a major health concern among older adults, leading to injury, reduced independence, and increased healthcare cost [1]. One of the current fall prevention methods for adults is reactive balance training (RBT), where individuals experience an intentional loss of balance and must complete steps to regain it to avoid the fall [2]. While land-based RBT has been shown to reduce fall risk, its accessibility is limited for some older adults due to fear of falling or injury, joint discomfort, and the physical burden of harness use [3]. Given the effectiveness of RBT, addressing its barriers is crucial to maximizing its reach and impact.

One method to overcome these barriers with land-based RBT might be to conduct the training in an aquatic environment. Water offers unique advantages, such as buoyancy and hydrostatic pressure, which can be used in RBT [4]. Water properties may enhance comfort and confidence. Beyond improving accessibility and comfort, aquatic reactive balance training (AquaReBal) may offer clinically meaningful advantages for older adults at risk of falls. The aquatic environment enables repeated exposure to balance perturbations while reducing joint loading and perceived threat associated with falling [5]. Water resistance, buoyancy, and multidirectional instability may also provide opportunities to challenge reactive stepping, postural control, and movement confidence [6]. As many older adults avoid land RBT due to fear, pain, or physical limitations, AquaReBal may represent a clinically relevant alternative approach for targeting reactive balance control and fall prevention.

Co-design has been defined as collaboration between researchers, end-users, and stakeholders to iteratively create tailored interventions [7]. Involving older adults in research allows them to develop new skills, fosters an inclusive research process [8] and enhance their participation in programs specifically designed for them [9]. However, it remains underutilized in older adult population [10,11].

In this study, we define Patient and Public Involvement (PPI) as a partnership between researchers and older adults, where both groups work together to make decisions, learn from each other, and co-create the program. Incorporating PPI in our study is grounded in participatory health research theories, which support involving end-users in developing solutions.

The aim of this paper is to describe the co-design of an aquatic reactive balance training (AquaReBal) program for older adults and the impact of engaging older adults with relevant lived experience.

## Materials and methods

### Study design

This study employed a qualitative co-design approach grounded in Participatory Action Research and used different methods to generate insights and design the final AquaReBal program. A detailed description of the finalized AquaReBal intervention protocol, including session structure, exercise progression, perturbation delivery, therapist supervision, and safety procedures, has been published separately in the feasibility study protocol [12]. Collective decision-making and knowledge exchange among the involved stakeholders played a key role throughout this process. We used the Guidance for Reporting Involvement of Patients and the Public 2 (GRIPP2) to report the patient and public involvement activities in this study [13]. This study also adopted the principles of the Participatory Action Research (PAR) approach. PAR emphasizes the active involvement of stakeholders through ongoing collaboration with researchers, following an iterative cycle of fieldwork or practice, reflection, planning, research, and action [14,15], aligning closely with co-design methodologies [16]. The goal was to enable the intended end users of the intervention to make decisions about its implementation based on their needs and expectations, thereby achieving outcomes that reflect their perspectives and their lived experiences [17].

The suggestions gathered in each session guided the development of AquaReBal program by identifying facilitators and barriers to practice adherence, and helped us understand the level of difficulty perceived by the older adults for the intended exercises [18]. This is particularly relevant given that RBT is a challenging type of balance training in which participants are repeatedly exposed to balance perturbations and perform balance reactions to avoid falls [2]. The study was approved by the University Health Network Research Ethics Board (study ID: 24-6146). Informed consent was obtained from all participants prior to participation; written consent was collected and documented.

### Participants

The research team was created in February 2025 and included physiotherapists, rehabilitation researchers, and trainees with expertise in aquatic therapy, reactive balance training, falls prevention, and patient-oriented research. The professional backgrounds and experiences of the research team may have influenced interpretation of participant feedback and prioritization of intervention components. To support collaborative interpretation and reduce reliance on a single perspective, intervention decisions were discussed iteratively within the multidisciplinary team and reviewed with older adult partners across multiple stages of the co-design process.

Older adult partners, who were part of the research team, were recruited through existing community and research networks affiliated with the Toronto Rehabilitation Institute. Purposeful recruitment was used to identify older adults with lived experience relevant to the aims of the project, including experiences related to falls, balance concerns, physical activity, aquatic exercise, or reactive balance training. Inclusion criteria included being aged 65 years or older, ability to communicate in English, willingness to participate in online and practical co-design sessions, and having personal experience relevant to fall prevention or balance training. No formal exclusion criteria were applied beyond inability to safely participate in the planned sessions or pool-based activities. Three older adult partners were involved – three women over the age of 65 years and living independently in the community. All partners had experienced at least one fall within the previous year. Two had previously participated in a randomized controlled trial of land-based reactive balance training. One of the partners had experience in exercising in an aquatic environment, as she regularly attended weekly aquatic exercise sessions. One partner had no experience in aquatic or RBT, but was participating in a fall prevention exercise group, aligning with the protocol’s goals of reducing fall incidence. Partners were generally physically active and able to independently participate in online discussions and pool-based sessions. None of the partners used assistive devices. Formal measures of mobility status, aquatic confidence, and ethnicity were not systematically collected as part of this co-design study. In line with PPI terminology, the older adults engaged in this study are referred to as “partners” to reflect their active and collaborative role in shaping the intervention design, rather than their involvement as research participants.

### Co-design sessions

**Fig 1:**
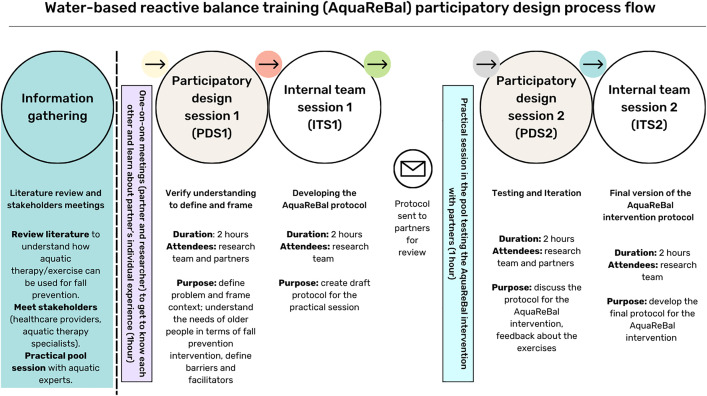
Co-design process.

The process followed a series of steps: initial engagement, relationship building, identification of concerns, participatory action, implementation, and subsequent reflection and evaluation [19]. Although the steps are described as separate, they often overlap and evolve dynamically throughout the process [20]. The process was structured into multiple iterative phases involving both the research team and partners (Fig 1). All meetings aside from the practical sessions in the pool were conducted online through MS Teams.

Initially, an information-gathering phase was conducted to inform the development of the AquaReBal program. This phase included a targeted literature review and stakeholder consultations with healthcare professionals and aquatic therapy specialists. The literature review aimed to identify current evidence related to the use of perturbation-based activities in aquatic environments. Relevant peer-reviewed articles were identified through searches of rehabilitation- and health-related databases and were used to inform discussions regarding potential exercise components, safety considerations, progression principles, and feasibility of implementing reactive balance training in water. Stakeholder consultations included online discussions and practical pool-based sessions with international aquatic therapy experts and researchers with expertise in land-based reactive balance training. Discussions focused on key principles of aquatic exercise, potential advantages and challenges of delivering perturbations in water, safety considerations, participant comfort, and possible exercise progressions. During practical sessions, experts explored different methods of delivering perturbations and discussed how water properties such as buoyancy and resistance could influence balance reactions and training intensity.

Insights gathered during this information-gathering phase informed the initial design of the AquaReBal intervention. Specifically, the findings contributed to the preliminary selection of exercises, perturbation approaches, safety procedures, and environmental considerations that were later refined through the participatory design process. Consultations were conducted with experts in land-based RBT, who provided insights into the key mechanisms, safety considerations, and progressions involved in delivering interventions. Importantly, the AquaReBal program is conceptually based on the Toronto Perturbation-Based Balance Training framework, which emphasizes task-specific, progressively challenging perturbations to improve reactive balance control and reduce fall risk.

To get to know the older adult partners and build trust, one-on-one meetings were conducted online by the principal researcher with each partner. The conversation included the purpose of the project, steps involved, and their role; their experiences with physical activity, including aquatic exercise; and their perceptions of balance, falls, and reactive balance training on land.

Following this, the first participatory design session (PDS1) was held with the research team and older adult partners. The session was conducted online using structured group discussions, collaborative brainstorming activities, and visual mapping tools (Miro board) to facilitate idea generation and shared reflection. The session aimed to collaboratively frame the problem, explore the needs and preferences of older adults regarding reactive balance training in water, and identify potential barriers and facilitators to the intervention. Older adult partners actively contributed their lived experiences, preferences, and suggestions throughout the discussions, and their input informed decision-making related to intervention design, participant experience, and safety considerations.

Insights from this session were synthesized by the research team and discussed during the first internal team session (ITS1), during which preliminary decisions regarding exercise selection, perturbation approaches, and session structure were made. Based on these discussions, a draft protocol for the practical pool session was developed and shared with the older adult partners one week prior to the pool session for review and feedback.

Practical sessions were conducted individually with each partner in the pool to test the proposed exercises and perturbation approaches. The practical sessions were conducted by two physiotherapists (one in the water leading the session and one on land taking notes and registering what was done). The sessions took place at the Toronto Rehabilitation Institute. The pool is situated on the second floor, with easy access via elevators and stairs. Pool sessions involved experiential testing and reflective discussion between the physiotherapists and older adult partners regarding feasibility, comfort, safety, perceived challenge, and suitability of the exercises. After each exercise, perceived intensity was recorded using the Balance Intensity Scale (BIS) [21].

Based on pool sessions, a second participatory design session (PDS2) was conducted involving both researchers and partners. The session included structured reflection and collaborative discussion regarding the pool experience, equipment, environmental considerations, and potential modifications to the intervention. Suggestions generated during the discussion were reviewed collectively and incorporated into subsequent refinements of the intervention. A second internal team session (ITS2) was then conducted, during which the research team finalized the AquaReBal protocol by integrating feedback and recommendations gathered across all prior phases of the co-design process.

All online sessions were audio-recorded and documented with notes to support analysis and ensure transparency and rigor in the co-design process. This multi-step, collaborative methodology aligns with recommendations for involving end users in the design of health interventions to enhance their relevance, usability, and potential for implementation [22]. Data gathered from participatory sessions, surveys, field notes, and practical pool observations were reviewed using an inductive qualitative content analysis approach. Members of the research team iteratively reviewed session recordings, written notes, and feedback to identify recurring ideas, concerns, facilitators, barriers, and recommendations related to the intervention design and participant experience. Emerging themes were discussed collaboratively within the research team and used to guide subsequent planning and refinement of the AquaReBal intervention. Insights generated during each phase directly informed concrete design decisions, including modifications to perturbation delivery, equipment selection, environmental adaptations, safety considerations, exercise progression, and participant-facing materials.

The co-design process reflected PAR principles through iterative cycles of reflection, planning, action, and refinement. Information gathered during the initial literature review, stakeholder consultations, and participatory discussions informed planning of practical pool sessions and intervention components. Feedback and observations obtained during action phases, including practical testing sessions, were subsequently reflected upon collaboratively by researchers and older adult partners to refine and adapt the intervention in subsequent stages.

## Results

In this section, we present the multi-step co-design process used to develop the AquaReBal program. After each step, feedback was gathered and reviewed in detail. The insights and recommendations informed specific modifications. The results are organized according to the sequence of activities conducted.

### Information gathering

There were only two other studies that used perturbations in the water as a part of the program [23,24]. An online meeting was held with international aquatic therapy experts from US, Canada, Australia and Europe (n = 7) which led to agreement between the experts about possible use of aquatic environment for RBT. This was followed by a practical session in the pool with the aquatic therapy experts (n = 4) which allowed to test first ideas of the tasks in the water and showed that despite water properties there is a possibility of performing quick reactions in the water.

### One-on-one meetings

Partners engaged in a range of physical activities and motivations included maintaining health, preventing physical decline, improving balance, and fostering social connections. Experiences with aquatic exercise were mixed. While some partners described it as enjoyable and socially engaging others reported barriers such as discomfort in locker rooms, lack of feedback, and cold or slippery environments. Concerns about balance and falling were prevalent, often tied to past experiences with falls. Fear of falling, particularly backward, was a recurring theme. Experiences with land-based RBT were diverse. One partner found the unpredictability stimulating, while the other experienced anxiety despite safety equipment. Notably, one partner reported improved balance confidence and responses to slips after land-based RBT. These insights directly shaped the development of AquaReBal, emphasizing safety, progressive challenge, and personalized support within an aquatic setting.

**Fig 2:**
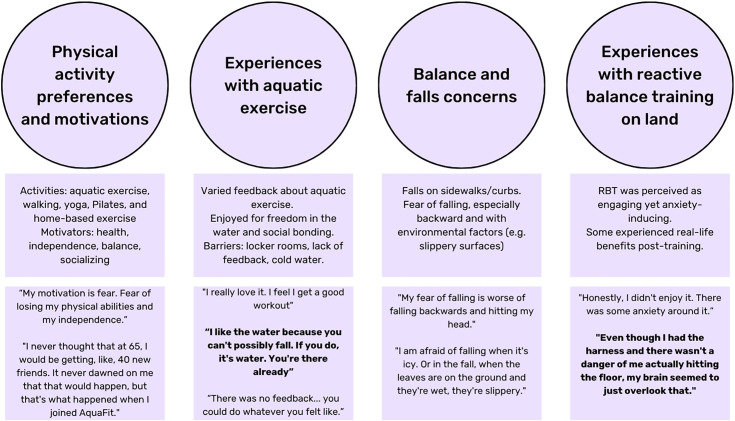
Lived experience context from the one-on-one meetings. Quotes bolded are to highlight the feelings regarding aquatic environment and the experiences with land-based RBT.

See Fig 2 for key themes and quotes from these initial one-on-one meetings with partners (Fig 2).

### Participatory design session 1

The meeting was attended by the principal researcher, two trainees, three older adult partners, one land-based RBT expert, and one aquatic therapy expert (n = 8). The meeting generated insights about the intervention as well as ideas to create a more inclusive and welcoming program, including the development of an ‘Introductory package’ for participants.

Motivations for taking part in aquatic interventions in general were mainly in the areas of health and functional well-being, social engagement, experience of being in the aquatic environment and the influence of the instructors. Partners emphasized the important role of the instructor. According to them, a strong connection with the instructor enhances motivation, enjoyment, and continued participation.

*“If you enjoy the people that you’re with, like the instructor and your co-participants, that it could lift your mood as well.” (Partner 3)*

*“I think the instructor is very important because they’re the ones that are motivating you to work harder, and if you don’t have that connection you won’t wanna go because it’s no longer fun.” (Partner 2)*

**Fig 3:**
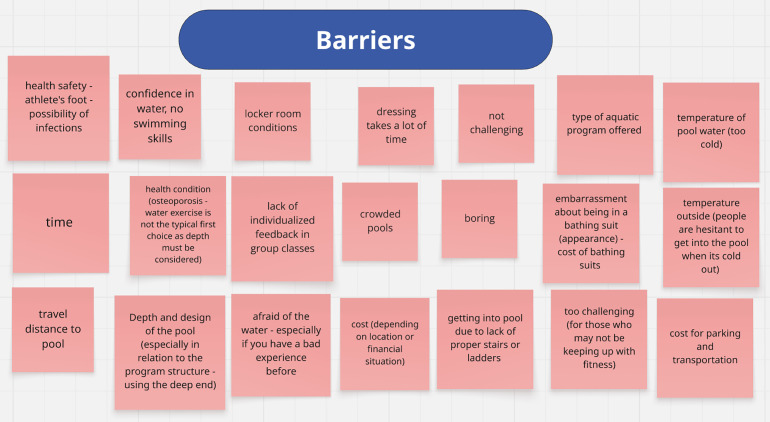
Miro board with the barriers to aquatic exercise discussed in the first participatory design session.

Barriers discussed focused on five main categories that can limit participation in aquatic exercise (Fig 3): health and physical safety concerns, comfort and confidence in the water, program design and suitability, practical and logistical barriers, and financial barriers. One partner mentioned how past negative experiences, even from early life, can have a lasting impact on willingness to engage in water-based programs.

The PDS1 meeting was evaluated in an anonymous way with a post-meeting survey (n = 7) to gather feedback. One person did not complete the survey. The respondents were overall very satisfied with the meeting based on the scoring (4.8/5). Respondents enjoyed meeting everyone involved in the project, and they found the input from other stakeholders meaningful, especially when it was grounded in their clinical experience and connected to participants’ real-world needs. Everyone reported that their ideas were heard during the session and brainstorming was seen as a valuable part of the meeting.

### Internal team session 1

The first internal team session included research team members (n = 5). The research team discussed insights gathered through the Miro board. Decisions were made to refine the ‘Introductory package’ by choosing the specific videos, clarify session instructions about pool depth and no swimming skills required, and adjust proposed exercises to better accommodate comfort, safety, and engagement. Protocol for the pool session was created and sent to the partners.

### Practical pool sessions

The activities tested with older adult partners included walking tasks (forward, backward, tandem walking, and walking in place), performed at different depths and with eyes closed or modified arm positions to increase task complexity. Lean-and-release was performed in forward, backward, and lateral directions, both in shallow and deeper areas of the pool. Additional perturbations were delivered using equipment, including multidirectional pulls with a waist belt during standing, walking, and rapid stepping, as well as manual pushes and pulls. In order to assess if the exercise is challenging the balance we used Balance Intensity Scale for each task. Partners reported moderate to high level of challenge of the exercises performed. One partner found the waist belt not comfortable for delivering perturbations.

### Participatory design session 2

**Fig 4:**
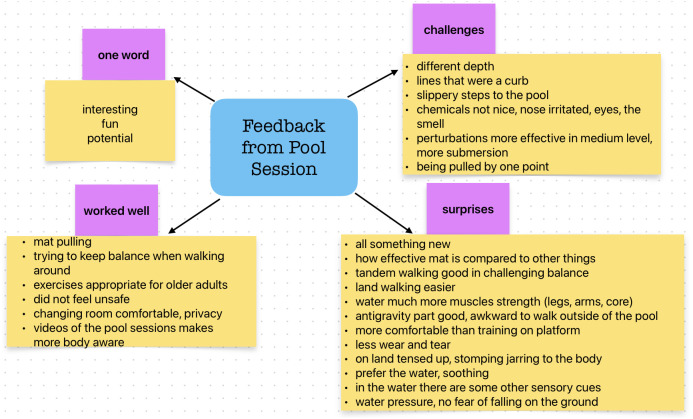
Summarized feedback from the partners after the pool session.

The meeting was attended by the principal researcher, two trainees, three older adult partners, one land-based RBT expert, and one aquatic therapy expert (n = 8). This session was focused on summarizing the feedback after the pool session from all the partners (Fig 4).

The aquatic environment was noted to reduce physical strain, increase muscle engagement due to water resistance, and enhance sensory input.

*“I think they were all more comfortable than the land on the platform that I find the platform is kind of jarring, you know, because you’re anticipating something horrible coming, which doesn’t necessarily happen, but you’re anticipating it” (Partner 1)*

This statement was made comparing AquaReBal to perturbations provided by a moving platform, experienced by one participant during their participation in an RBT clinical trial. Regarding the AquaReBal exercises, partners felt they were appropriate and safe for older adults. A recurring theme was related to comfort and safety of perturbation delivery. Partners reported discomfort with the hip belt, which led the research team to consider vest-based perturbation approaches for future iterations of the intervention. The mat was considered effective for creating balance challenges, whereas the pool noodle was viewed as less effective.

*“I didn’t particularly like being pulled from one point and we discussed that a vest might be better because then it’s kind of pulling your whole body, not just one part of your hip” (Partner1)*

The pool’s depth presented challenges as the changes of the depth were unclear and confusing. Partners recommended more distinct physical markers or the use of a pool with a gradual slope. Concerns were also raised about the chemicals used in the pool as one partner experienced irritation.

Partners described AquaReBal as interesting, enjoyable and promising for future use.

*“Fun. Interesting. Potential.” (Partner 3)*

Based on partner input, we collaboratively developed materials for the ‘Introductory package’ to be included in an initial email to new participants (Table 1). These included a checklist of items to bring to the sessions, photographs of the pool facilities, a short video demonstrating example exercises. The content was informed by the partners’ own experiences and reflected the resources they felt would have made their initial participation in the study easier.

**Table 1:** Items included in the introductory package.

Item	Description
**Introductory email content**	The email included some additional information which will be helpful for the first session in the pool: - private change room, including toilet, sink, and shower - there will always be staff person in the pool with you - lockers are available to store personal items - the pool has 3 levels with the deepest being 1.35m (4’-5”). - it’s unlikely your head will be under water at any time during the exercises - ability to swim is not required
**Changing room**	Changing rooms are private and include a toilet, sink, and shower. Lockers are available outside for storing belongings, though items may also be kept in the pool area during the session, making locker use optional.
**Therapy pool**	The pool is accessible by stairs with a handrail. The pool is divided into three depth levels, allowing exercises to be adapted to individual needs, abilities, confidence in the water, and the level of difficulty imposed by each depth.
**Check list: items to bring to the training sessions**	A checklist with essential items to bring to the training session, such as a flipflops, soap and other personal necessities to ensure safety, hygiene and comfort before and after each session.
**Video Showing Exercises**	A video showcasing a few exercises that will be used in the pool sessions to give participants an idea of what to expect. The video also includes some of the equipment to be used during the sessions such as the mat. https://www.youtube.com/watch?v=qEyA2gh0ZGE

The meeting was evaluated in an anonymous way with a post-meeting survey (n = 6) to gather feedback. Two people did not complete the survey. The meeting was overall assessed positively (4.6/5). People valued the active engagement of meeting attendees, the opportunity to share feedback and reflections, and the focused discussion on aspects of the intervention, such as what worked better or worse for perturbations. The structured format was also appreciated, as it allowed everyone to contribute. Suggestions for improvement included shortening the session, providing discussion questions in advance to allow for more considered responses, and offering a brief overview of study eligibility criteria, goals, and objectives to ensure feedback was well aligned with the research aims. All respondents reported that their ideas were heard and considered.

### Internal team session 2

During the second internal team session (n = 7) the research team made specific adjustments including modifications to perturbation delivery and refinements to session progression to optimize safety and challenge (Table 2). Outcomes of this session were directly applied to finalize the AquaReBal program ensuring the intervention was responsive to user experiences.

**Table 2:** Summary of changes made based on the feedback from partners.

Theme	Feedback from partners	Resulting modification to AquaReBal
Safety and comfort	Concerns regarding discomfort and perceived pulling force of the hip belt	Exploration of vest-based perturbation delivery methods
Fear and confidence	Aquatic environment perceived as less threatening compared to land-based perturbations	Emphasis on gradual progression and supportive therapist guidance
Pool environment	Difficulty identifying pool depth transitions	Additional orientation information and visual explanations included in participant materials
Participant preparation	Uncertainty regarding what to expect during sessions	Development of introductory package with photos, checklist, and exercise video
Instructor support	Importance of encouragement, reassurance, and communication from instructors	Greater emphasis on therapist-participant interaction and support during sessions
Exercise challenge	Some exercises perceived as more effective and engaging than others	Refinement of exercise selection and perturbation progression
Accessibility and comfort	Concerns regarding locker rooms, chemicals, and pool logistics	Additional practical information included before participation

## Discussion

This study describes the co-design process for developing the AquaReBal program, a novel aquatic RBT intervention for older adults. By including stakeholders’ perspectives from the start, we aimed to build a program that reflects their real-world experiences while improving accessibility, especially for those facing barriers with traditional land-based balance training. This study adds to the growing research on participatory and user-centered design by highlighting the active role older adults can play in creating interventions.

The co-design approach used in this study not only informed the development of AquaReBal but also highlighted the feasibility of engaging older adults in meaningful ways throughout the research process. Team members and partners reported high satisfaction with participatory sessions, felt their input was valued, and contributed to shaping both the intervention content and delivery. This supports the growing movement toward PPI in rehabilitation research, which has been associated with improved relevance, uptake, and sustainability of interventions [25].

Building on international guidance for reporting patient and public involvement – the GRIPP2 framework [13], our study illustrates the value of transparent reporting of co-design processes in rehabilitation research. In line with GRIPP2, we documented the aims of involving older adults, the methods of engagement, and the outcomes of involvement. Contextual factors, such as partners’ previous experience with aquatic exercise and the supportive facilitation of researchers, positively influenced the process. Importantly, the involvement of older adults not only shaped practical elements of AquaReBal but also contributed to theory development by demonstrating how co-design in aquatic environments can extend models of participatory intervention design. Transparent reporting of these processes is critical for building a cumulative evidence base and will support replication and scaling of participatory aquatic interventions in future research [26].

Our findings highlight several key considerations for developing aquatic interventions. First, participants emphasized the importance of safety, comfort, and confidence in the water. This aligns with prior research indicating that fear of falling and environmental barriers are significant determinants of older adults’ participation in aquatic exercise programs [6]. The use of water as a medium for RBT appeared to mitigate some of the physical strain and joint discomfort associated with land-based perturbations, supporting previous evidence that aquatic environments provide a safe and supportive context for balance training in older adults [4]. From a clinical perspective, AquaReBal may address an important gap in fall prevention rehabilitation by providing an alternative format for delivering RBT to individuals who may not tolerate or engage with land-based approaches. The ability to safely expose older adults to repeated balance perturbations in a supportive aquatic environment may facilitate participation among individuals with fear of falling, joint discomfort, reduced confidence, or mobility limitations. Although the present study did not evaluate intervention effectiveness, the co-design process identified several features that may enhance adherence, acceptability, and accessibility, which are important considerations for successful implementation of fall prevention interventions in clinical practice. Social and relational aspects were also consistently identified as motivating factors. Partners highlighted the role of instructors in fostering engagement, enjoyment, and continued participation. This is consistent with research showing that instructor support, feedback, and social interaction can enhance adherence to exercise programs among older adults [27].

Practical and logistical considerations, including equipment, pool layout, and environmental cues, were critical for optimizing safety and usability. Feedback on the hip belt versus vest for perturbations and recommendations for pool depth demonstrate the importance of iterative, participatory design in adapting interventions to the real-world needs of older adults. This reinforces the value of including end-users in program development, ensuring that interventions are acceptable, and aligned with older adults’ preferences [28].

Older adult partners played an active role in shaping key aspects of the AquaReBal protocol, including perturbation delivery methods, environmental adaptations, participant-facing materials, and considerations related to comfort and safety. Decision-making occurred iteratively throughout the project, with researchers and older adult partners collaboratively reviewing experiences from discussions and practical pool sessions to refine intervention components. While no major disagreements emerged during the process, balancing participant preferences with feasibility, safety considerations, and practical limitations of the aquatic environment required ongoing discussion and negotiation within the research team. The study also highlighted the importance of creating supportive and flexible environments that enable meaningful involvement of older adults in rehabilitation research. Factors such as trust-building, clear communication, opportunities for reflection, and responsiveness to participant feedback appeared to facilitate engagement throughout the co-design process.

While participants valued the opportunity to shape the intervention, challenges emerged in balancing diverse preferences, reconciling safety concerns with feasibility, and navigating logistical constraints of pool access and equipment availability. Such challenges mirror broader findings in the PPI literature that underline the need to acknowledge both benefits and limitations of involvement rather than presenting idealized accounts [29]. Contextual factors, such as the supportive facilitation provided by researchers, pre-existing trust with partners, and their prior exposure to aquatic exercise, facilitated meaningful engagement. At the same time, process-related factors, including the limited time available for iterative cycles and scheduling constraints, posed barriers to deeper collaboration.

Our work extends existing models of co-design by situating them in an aquatic environment, a context that has been underexplored in rehabilitation research. By embedding PPI from the earliest stages, AquaReBal contributes to the evidence base that co-design might generate not only more acceptable interventions but also novel insights into how balance training is experienced in specific settings.

### Limitations

The number of older adult partners involved in the co-design process was small and consisted only of women, which may limit the diversity and transferability of perspectives informing the intervention development. Additionally, partners were relatively active and may not fully represent older adults with higher levels of frailty, functional limitations, or different experiences and confidence levels related to aquatic environments. As this study focused on describing an initial co-design process, the findings should not be interpreted as representative of the broader older adult population. Environmental and logistical factors specific to the Toronto Rehabilitation Institute pool may not generalize to other settings. Finally, while participatory design provided rich qualitative insights, the effectiveness of AquaReBal in improving reactive balance control and reducing falls remains to be tested.

### Implications and future directions

The co-design methodology described here can serve as a model for developing other rehabilitation interventions that integrate end-user perspectives, environmental considerations, and evidence-based principles. The findings suggest that AquaReBal has the potential to be a safe, engaging, and adaptable program for older adults. Future research should evaluate the program’s feasibility, acceptability, and preliminary effectiveness in a larger, diverse cohort.

## Conclusions

In conclusion, the creation of AquaReBal suggests that co-design in the development of exercise interventions can enhance safety, enjoyment, and relevance, while addressing barriers to participation. The program provides a potentially promising approach for fall prevention, blending evidence-based principles of RBT with the unique advantages of the aquatic environment.

## References

[1] Vaishya R, Vaish A. Falls in older adults are serious. Indian J Orthop. 2020;54(1):69–74. doi: 10.1007/s43465-019-00037-x 32257019 PMC7093636

[2] Devasahayam AJ, Farwell K, Lim B, Morton A, Fleming N, Jagroop D, et al. The effect of reactive balance training on falls in daily life: an updated systematic review and meta-analysis. Phys Ther. 2022;103(1):pzac154. doi: 10.1093/ptj/pzac154 37651698

[3] Gerards MHG, Sieben J, Marcellis R, de Bie RA, Meijer K, Lenssen AF. Acceptability of a perturbation-based balance training programme for falls prevention in older adults: a qualitative study. BMJ Open. 2022;12(2):e056623. doi: 10.1136/bmjopen-2021-056623 35210345 PMC8883254

[4] Deng Y, Tang Z, Yang Z, Chai Q, Lu W, Cai Y, et al. Comparing the effects of aquatic-based exercise and land-based exercise on balance in older adults: a systematic review and meta-analysis. Eur Rev Aging Phys Act. 2024;21(1):13. doi: 10.1186/s11556-024-00349-4 38764039 PMC11102618

[5] Kutzner I, Richter A, Gordt K, Dymke J, Damm P, Duda GN, et al. Does aquatic exercise reduce hip and knee joint loading? In vivo load measurements with instrumented implants. PLoS One. 2017;12(3):e0171972. doi: 10.1371/journal.pone.0171972 28319145 PMC5358747

[6] Becker BE. Aquatic therapy: scientific foundations and clinical rehabilitation applications. PM R. 2009;1(9):859–72. doi: 10.1016/j.pmrj.2009.05.017 19769921

[7] Constantin N, Edward H, Ng H, Radisic A, Yule A, D’Asti A, et al. The use of co-design in developing physical activity interventions for older adults: a scoping review. BMC Geriatr. 2022;22(1):647. doi: 10.1186/s12877-022-03345-4 35941570 PMC9358386

[8] James H, Buffel T. Co-research with older people: a systematic literature review. Ageing Soc. 2022;43(12):2930–56. doi: 10.1017/s0144686x21002014

[9] McKay H, Nettlefold L, Bauman A, Hoy C, Gray SM, Lau E, et al. Implementation of a co-designed physical activity program for older adults: positive impact when delivered at scale. BMC Public Health. 2018;18(1):1289. doi: 10.1186/s12889-018-6210-2 30470209 PMC6251145

[10] Corrado AM, Benjamin-Thomas TE, McGrath C, Hand C, Laliberte Rudman D. Participatory action research with older adults: a critical interpretive synthesis. Gerontologist. 2020;60(5):e413–27. doi: 10.1093/geront/gnz080 31264680

[11] Hand C, Keber A, McFarland J, McGrath C, Laliberte Rudman D, Seale L, et al. Neighbourhood-based participatory action research with older adults: facilitating participation through virtual and remote methods. Methodol Innov. 2024;17(4):248–60. doi: 10.1177/20597991241292368

[12] Ogonowska-Slodownik A, Marinho-Buzelli A, Danells C, Musselman KE, Bonnyman A, Alavinia M, et al. Feasibility of Aquatic Reactive Balance Training (AquaReBal) for older adults: protocol for a single-arm pre-post study. Gerontology. 2026;72(5):375–83. doi: 10.1159/000550917 41779675 PMC13082763

[13] Staniszewska S, Brett J, Simera I, Seers K, Mockford C, Goodlad S, et al. GRIPP2 reporting checklists: tools to improve reporting of patient and public involvement in research. BMJ. 2017;358:j3453. doi: 10.1136/bmj.j3453 28768629 PMC5539518

[14] Cusack C, Cohen B, Mignone J, Chartier MJ, Lutfiyya Z. Participatory action as a research method with public health nurses. J Adv Nurs. 2018;74(7):1544–53. doi: 10.1111/jan.13555 29489024

[15] White GW, Suchowierska M, Campbell M. Developing and systematically implementing participatory action research. Arch Phys Med Rehabil. 2004;85(4 Suppl 2):S3-12. doi: 10.1016/j.apmr.2003.08.109 15083417

[16] Howard Z, Somerville MM. A comparative study of two design charrettes: implications for codesign and participatory action research. CoDesign. 2014;10(1):46–62. doi: 10.1080/15710882.2014.881883

[17] Tremblay M-C, Bradette-Laplante M, Bérubé D, Brière É, Moisan N, Niquay D, et al. Engaging indigenous patient partners in patient-oriented research: lessons from a one-year initiative. Res Involv Engagem. 2020;6:44. doi: 10.1186/s40900-020-00216-3 32760594 PMC7376932

[18] Harrison JD, Auerbach AD, Anderson W, Fagan M, Carnie M, Hanson C, et al. Patient stakeholder engagement in research: a narrative review to describe foundational principles and best practice activities. Health Expect. 2019;22(3):307–16. doi: 10.1111/hex.12873 30761699 PMC6543160

[19] Leask CF, Sandlund M, Skelton DA, Altenburg TM, Cardon G, Chinapaw MJM, et al. Framework, principles and recommendations for utilising participatory methodologies in the co-creation and evaluation of public health interventions. Res Involv Engagem. 2019;5:2. doi: 10.1186/s40900-018-0136-9 30652027 PMC6327557

[20] Bazzano AN, Martin J, Hicks E, Faughnan M, Murphy L. Human-centred design in global health: a scoping review of applications and contexts. PLoS One. 2017;12(11):e0186744. doi: 10.1371/journal.pone.0186744 29091935 PMC5665524

[21] Farlie MK, Keating JL, Molloy E, Bowles K-A, Neave B, Yamin J, et al. The balance intensity scales for therapists and exercisers measure balance exercise intensity in older adults: initial validation using rasch analysis. Phys Ther. 2019;99(10):1394–404. doi: 10.1093/ptj/pzz092 31309981 PMC6821236

[22] Slattery P, Saeri AK, Bragge P. Research co-design in health: a rapid overview of reviews. Health Res Policy Syst. 2020;18(1):17. doi: 10.1186/s12961-020-0528-9 32046728 PMC7014755

[23] Elbar O, Tzedek I, Vered E, Shvarth G, Friger M, Melzer I. A water-based training program that includes perturbation exercises improves speed of voluntary stepping in older adults: a randomized controlled cross-over trial. Arch Gerontol Geriatr. 2013;56(1):134–40. doi: 10.1016/j.archger.2012.08.003 22951028

[24] Muthukrishan R, Badr Ul Islam FM, Shanmugam S, Arulsingh W, Gopal K, Kandakurti PK, et al. Perturbation-based balance training in adults aged above 55 years with chronic low back pain: a comparison of effects of water versus land medium - a preliminary randomized trial. Curr Aging Sci. 2024;17(2):156–68. doi: 10.2174/0118746098254991231125143735 38111118

[25] Brett J, Staniszewska S, Mockford C, Herron-Marx S, Hughes J, Tysall C, et al. A systematic review of the impact of patient and public involvement on service users, researchers and communities. Patient. 2014;7(4):387–95. doi: 10.1007/s40271-014-0065-0 25034612

[26] Brown N. Scope and continuum of participatory research. Int J Res Method Educ. 2022;45(2):200–11. doi: 10.1080/1743727X.2021.1902980

[27] Morrison L, McDonough MH, Zimmer C, Din C, Hewson J, Toohey A, et al. Instructor social support in the group physical activity context: older participants’ perspectives. J Aging Phys Act. 2023;31(5):765–75. doi: 10.1123/japa.2022-0140 36948211

[28] Concannon TW, Meissner P, Grunbaum JA, McElwee N, Guise J-M, Santa J, et al. A new taxonomy for stakeholder engagement in patient-centered outcomes research. J Gen Intern Med. 2012;27(8):985–91. doi: 10.1007/s11606-012-2037-1 22528615 PMC3403141

[29] Agyei-Manu E, Atkins N, Lee B, Rostron J, Dozier M, Smith M, et al. The benefits, challenges, and best practice for patient and public involvement in evidence synthesis: a systematic review and thematic synthesis. Health Expect. 2023;26(4):1436–52. doi: 10.1111/hex.13787 37260191 PMC10349234

