# Supplementary material for: Using a co-design approach to develop aquatic reactive balance training for fall prevention
Source: PLoS One. 2026 Jul 15;21(7):e0345435. doi: 10.1371/journal.pone.0345435 (PMC13372110; doi:10.1371/journal.pone.0345435)
Supplement: Attachment — Submitted filename: PONE-D-26-11038.docx [file pone.0345435.s001.docx]

*Using a co-design approach to develop aquatic reactive balance training for fall prevention*

Overall Recommendation

Major Revision

This manuscript presents a timely and relevant study describing the co-design of an aquatic reactive balance training (AquaReBal) intervention for older adults. The topic is novel and clinically meaningful, particularly given the increasing interest in participatory rehabilitation research and fall prevention strategies for older adults. The integration of patient/public involvement (PPI) and aquatic therapy concepts is a notable strength.

However, the manuscript currently reads more as a descriptive project report than a rigorously structured scientific study. Several methodological, reporting, and analytical aspects require clarification and strengthening before the work is suitable for publication in PLOS ONE.

General Comments

Strengths

1. Novelty and relevance
   - The concept of aquatic reactive balance training is innovative and clinically relevant for older adults with fear of falling or intolerance to land-based perturbation training.
2. Strong patient/public involvement
   - The manuscript appropriately integrates older adult partners throughout the intervention development process.
   - Use of the GRIPP2 framework is commendable.
3. Clear participatory workflow
   - The iterative co-design process is well organized and visually represented in Figure 1.
4. Practical rehabilitation relevance
   - The paper provides useful implementation insights for clinicians developing aquatic rehabilitation programs.

Major Concerns

1. Lack of rigorous qualitative methodology

The manuscript describes feedback collection through meetings, surveys, and observations, but it does not clearly describe:

- how qualitative data were analyzed,
- whether thematic analysis/content analysis/framework analysis was used,
- who coded the data,
- whether coding was inductive or deductive,
- how trustworthiness/rigor was ensured.

Currently, the Results section largely summarizes discussions narratively rather than presenting a formal qualitative analysis.

Recommendation

The authors should:

- explicitly describe the qualitative analytical approach,
- explain coding procedures,
- clarify how themes/categories were generated,
- include strategies for credibility and rigor (e.g., triangulation, reflexivity, audit trail, member checking).

Without this, the study lacks methodological transparency expected for qualitative/co-design research.

2. Sample size and representativeness are very limited

Only three older adult partners participated, all of whom were women and relatively active.

This substantially limits:

- transferability,
- diversity of perspectives,
- applicability to frailer populations,
- gender representation.

While the limitation is acknowledged, the manuscript tends to overstate broader applicability.

Recommendation

The discussion and conclusions should be more cautious regarding generalizability and implementation potential.

3. Insufficient detail regarding the final AquaReBal protocol

The paper describes the co-design process but does not adequately describe the final intervention itself.

Missing details include:

- session duration,
- frequency,
- total intervention dosage,
- perturbation intensity progression,
- therapist-to-participant ratio,
- safety monitoring procedures,
- exact perturbation methods,
- training goals.

For replication and implementation, readers need a clearer description of the finalized protocol.

Recommendation

Consider adding:

- a supplementary table outlining the finalized AquaReBal protocol,
- exercise progression principles,
- safety criteria and stopping rules.

4. Limited integration of co-design theory

Although participatory action research and co-design are discussed, the manuscript remains largely descriptive and does not critically engage with:

- co-design theory,
- power-sharing dynamics,
- participatory rehabilitation frameworks,
- challenges of stakeholder integration.

The Discussion would benefit from deeper theoretical interpretation.

Recommendation

Expand discussion regarding:

- how older adults influenced decision-making,
- whether any disagreements occurred,
- how consensus was achieved,
- lessons learned about participatory rehabilitation design.

5. Data availability statement inconsistency

There is a discrepancy between the submission metadata and manuscript text.

The submission form states:

“All relevant data are within the manuscript and its Supporting Information files.”

However, the manuscript states:

“The data that support the findings of this study are available from the corresponding author upon reasonable request.”

PLOS ONE requires consistency and generally discourages “available on request” statements unless justified.

Recommendation

Revise the data availability statement to comply fully with PLOS ONE policy.

Minor Concerns

1. Language and grammar issues

Several sentences require proofreading.

Examples:

- “to to shape the program”
- “water environment” should often be “aquatic environment”
- inconsistent use of “partners,” “participants,” and “older adults”

A professional language edit is recommended.

2. Figures need improvement

Some figures appear visually low-resolution and difficult to interpret:

- Figure 3
- Figure 4

The Miro/Freeform screenshots look informal for journal publication.

Recommendation

Convert figures into professionally formatted thematic diagrams/tables.

3. Limited demographic reporting

The manuscript provides minimal participant characteristics.

Recommendation

Include:

- age range,
- ethnicity,
- mobility status,
- assistive device use,
- fall history,
- aquatic confidence/swimming ability.

4. Reflexivity statement missing

Given the participatory nature of the study, the manuscript should discuss:

- researchers’ positionality,
- prior relationships with participants,
- potential influence on data interpretation.

5. Need clearer distinction between feasibility and intervention efficacy

At times, the manuscript implies therapeutic benefit despite no outcome evaluation.

Recommendation

Avoid efficacy-oriented language unless formally tested.

For example:

- “demonstrating potential for broader implementation”
  may overstate findings.

Section-Specific Comments

Abstract

The abstract is clear overall but should mention:

- the qualitative/co-design analytical approach,
- the small sample size as context.

Introduction

Strong rationale overall. However:

- the distinction between aquatic therapy and aquatic reactive balance training should be more explicitly defined,
- stronger mechanistic rationale for why aquatic perturbation training may improve reactive balance would strengthen the paper.

Methods

This section requires the most improvement.

Please clarify:

- recruitment strategy,
- inclusion/exclusion criteria,
- qualitative analysis procedures,
- survey questions/scales,
- documentation methods,
- decision-making process for incorporating feedback.

Results

The Results section currently mixes:

- descriptive reporting,
- interpretation,
- participant quotes.

Consider reorganizing into themes/subthemes.

A thematic table summarizing:

- barriers,
- facilitators,
- design recommendations,
  would substantially improve readability.

Discussion

The Discussion is generally appropriate but should:

- critically discuss limitations of participatory co-design,
- better integrate qualitative methodology literature,
- avoid overclaiming intervention readiness.
