# Supplementary material for: Using a co-design approach to develop aquatic reactive balance training for fall prevention
Source: PLoS One. 2026 Jul 15;21(7):e0345435. doi: 10.1371/journal.pone.0345435 (PMC13372110; doi:10.1371/journal.pone.0345435)
Supplement: Attachment — Submitted filename: Response to Reviewers.docx [file pone.0345435.s003.docx]

**Response to Reviewers’ Comments**

**“Using a co-design approach to develop aquatic reactive balance training for fall prevention”**

Thank you for the review of our manuscript. We sincerely appreciate the time, expertise, and constructive feedback provided by the Editor and Reviewers. We carefully considered all comments and revised the manuscript accordingly. We believe the revisions have substantially strengthened the methodological clarity, reporting transparency, and overall quality of the manuscript. All changes made in the revised manuscript are highlighted in red text. Below, we provide a detailed point-by-point response to each comment

**Editorial comments**

**1) Grant information discrepancy**

**Comment:**
We note that the grant information provided in the ‘Funding Information’ and ‘Financial Disclosure’ sections do not match.

**Response:**
Thank you for identifying this inconsistency. We carefully reviewed and corrected the funding information to ensure consistency across all submission materials and manuscript sections.

**2) Role of the funder statement**

**Comment:**
Please include the amended Role of Funder statement in your cover letter.

**Response:**
Thank you. The requested Role of Funder statement has been added to the cover letter as requested.

**3) Ethics statement placement**

**Comment:**
Your ethics statement should only appear in the Methods section.

**Response:**
Thank you for this clarification. The ethics statement appears only in the Methods section.

**4) Data availability statement**

**Comment:**
Please ensure compliance with PLOS ONE data availability policy.

**Response:**
The submission contains all raw data required to replicate the results of the study.

**Reviewer #1**

**Comment:** Among the research participants, there were only 3 elderly individuals, all of whom were female and thus not representative.

**Response:** We thank the reviewer for this important observation. We would like to clarify that the older adults involved in this study were engaged as partners in the co-design process rather than as research participants in an intervention study. Their role was to collaboratively contribute lived experience perspectives to the development and refinement of the AquaReBal program. Nevertheless, we acknowledge that the small number of older adult partners and the inclusion of only women limit the diversity and transferability of perspectives represented in the co-design process. We therefore revised the Limitations section to more explicitly discuss the limited gender representation, the relatively active profile of the partners, and the potential lack of applicability to older adults with greater frailty, different cultural backgrounds, or differing experiences with aquatic exercise. We also clarified that this study aimed to describe an initial co-design process rather than provide representative perspectives of the broader older adult population.

**Comment:** The key information such as the training structure, frequency, duration, action composition, and safety standards of the final solution of AquaReBal is not clearly presented.

**Response:** Thank you for this valuable suggestion. We agree that clear reporting of the finalized AquaReBal protocol is important for reproducibility and implementation. To address this comment, we added a citation to our recently accepted protocol paper, which provides detailed information regarding the AquaReBal intervention structure, session frequency and duration, exercise progression, perturbation delivery, therapist supervision, and safety procedures:

Ogonowska-Slodownik A, Marinho-Buzelli A, Danells C, Musselman K, Bonnyman A, Alavinia M, Mansfield A. *Feasibility of aquatic reactive balance training (AquaReBal) for older adults: protocol for a single-arm pre-post study*. Gerontology 2026 Mar 4:1-9. doi: 10.1159/000550917.

We also added a brief statement in the manuscript (methods, study design) directing readers to this publication for the full intervention protocol details.

**Reviewer #2**

**Introduction**

**Comment:** Introduction is lengthy with repetition of the main idea.

**Response:** Thank you for this suggestion. The Introduction was revised to improve clarity and conciseness. Repetitive statements were removed, and the rationale was streamlined while maintaining the key theoretical and clinical context.

**Comment:** I am not sure why the authors refer to participating older adults as “partners”. It might be a cultural thing, but it is confusing. Please modify to participants.

**Response:** Thank you for this comment. We understand the concern regarding terminology. However, we intentionally retained the term “partners” throughout the manuscript because it aligns with patient and public involvement (PPI) terminology and reflects the collaborative role older adults played in the co-design process rather than their role as passive study participants. To improve clarity, we added an explanation of this terminology in the methods.

**Comment:**Authors need to declare how their recruitment process went. How did they choose their older adult participants? What were their inclusion and exclusion criteria? Every detail matters.

**Response:** Thank you. We expanded the Methods section to clearly describe the recruitment.

**Comment:** Authors did good preparatory steps with consultation of experts related to RBT and aquatic therapy to make an evidence-based intervention. And while the idea of co-designing the program with the recipients in mind is indeed promising. Including very few older adult participants (only 3 women) made the results reflect a mere personal opinion rather than becoming reflective of a group of older adults and limits the value of the work done. Including more older adults and analyzing their sociodemographic and functional levels or adding baseline and post-intervention functional levels of the participants could have added a greater value to this work.

**Response:** We thank the reviewer for this important observation. We would like to clarify that the older adults involved in this study were engaged as partners in the co-design process rather than as research participants in an intervention study. Their role was to collaboratively contribute lived experience perspectives to the development and refinement of the AquaReBal program. Nevertheless, we acknowledge that the small number of older adult partners and the inclusion of only women limit the diversity and transferability of perspectives represented in the co-design process. We therefore revised the Limitations section to more explicitly discuss the limited gender representation, the relatively active profile of the partners, and the potential lack of applicability to older adults with greater frailty, different cultural backgrounds, or differing experiences with aquatic exercise. We also clarified that this study aimed to describe an initial co-design process rather than provide representative perspectives of the broader older adult population.

**Comment:** Authors start the results section with a section related to how information gathering process proceeded. Any information related to the process of the design itself belongs to the methods section. The findings of each step of that process belongs to the results. Please rearrange accordingly.

**Response:** Thank you for this helpful suggestion. We reorganized the manuscript to improve separation between procedural descriptions and findings. Information regarding how the co-design process was conducted was moved to the Methods section, while findings remain in the Results section.

**Comment:** Participants should be coded with numbers.

**Response:** Thank you. Participant quotes are now coded consistently (e.g., Partner 1, Partner 2, Partner 3) throughout the manuscript.

**Comment:** Authors mentioned using Balance Intensity Scale to measure perceived intensity in the results section. Please add any used measurement tool to the methods section as well.

**Response:** Thank you for noting this omission. Information regarding the Balance Intensity Scale (BIS), has now been added to the Methods section.

**Reviewer #3**

**Comment:** The study design is not sufficiently or explicitly stated. The authors should clearly define the study design in a single, concise sentence early in the section (e.g., “This study employed a qualitative co-design approach grounded in Participatory Action Research”). The current description combines conceptual frameworks without clearly positioning the study within a recognized methodological category.

**Response:** Thank you for this valuable comment. We revised the opening of the Methods section to clearly state the study design.

**Comment:** While the authors provide a detailed narrative of the co-design process (including participatory design sessions, internal team sessions, and practical testing), the methodological description remains incomplete and lacks sufficient clarity and rigor from a research design perspective.
First, although an initial information-gathering phase is mentioned (literature review and stakeholder meetings), this phase is not adequately described. There is no information on:
- how the literature review was conducted,
- how stakeholder meetings were structured,
- how data from this phase informed subsequent steps.

**Response:** Thank you for this important comment. We agree that the initial information-gathering phase required further clarification. We expanded the Methods section to provide additional details

**Comment:** Second, the co-design process itself is presented as a sequence of activities (PDS1, ITS1, practical sessions, PDS2, ITS2), but important methodological details are missing. Specifically:
- The methods used within the participatory sessions are not clearly described (e.g., whether structured discussions, focus groups, workshops, or specific co-design techniques were used).
- The role of participants (e.g., level of decision-making power vs. advisory input) is not explicitly defined.

**Response:** Thank you for this important comment. We agree that additional methodological detail regarding the co-design sessions improves the clarity and rigor of the manuscript. We revised the Methods section to more clearly describe the structure and facilitation of the participatory design sessions

**Comment:** Third, although sessions were audio-recorded and supported by notes, there is no clear description of how the data were analyzed. It is unclear:
- whether a qualitative analysis was conducted,
- which analytic approach was used (e.g., thematic analysis, content analysis),
- how insights were translated into concrete design decisions.
Finally, the link between the stated theoretical framework (Participatory Action Research) and the actual procedures is not fully demonstrated. While iterative cycles are implied, the manuscript does not explicitly map the described steps onto PAR phases (e.g., reflection, planning, action).

**Response:** Thank you for this important comment. We agree that the manuscript required clearer description of the analytical process and stronger alignment between the Participatory Action Research (PAR) framework and the study procedures. We revised the Methods section.

**Comment:** The manuscript clearly emphasizes the value of co-design and participatory approaches; however, the claimed contribution appears to be predominantly methodological rather than clinical. Both in the Introduction and Discussion, the study’s relevance is framed mainly in terms of advancing co-design practices, rather than demonstrating its added value for rehabilitation outcomes.
While these aspects are valuable, they are not sufficiently connected to the clinical significance of the AquaReBal intervention, particularly in terms of expected impact on fall prevention, improvements in balance or functional outcomes, and added value compared to existing rehabilitation approaches. As a result, the manuscript risks being perceived primarily as a process paper, with limited evidence of clinical innovation or advancement.

**Response:** Thank you for this thoughtful comment. We agree that the clinical significance of AquaReBal required stronger emphasis throughout the manuscript. In response, we revised the Introduction and Discussion to better articulate the potential rehabilitation relevance of aquatic reactive balance training,

**Comment:** In contrast, the results of the co-design process itself are underdeveloped and insufficiently reported. There is limited detail on the specific suggestions made by participants, the nature of the feedback is not clearly categorized, and the concrete changes made to the intervention based on participant input are not systematically presented.
This limits the reader’s ability to understand what was learned from participants, how their contributions shaped the intervention, and the added value of the participatory approach.

**Response:** Thank you for this important and constructive comment. We agree that the distinction between procedural descriptions and findings required further clarification. In response, we substantially revised and reorganized both the Methods and Results sections. Procedural and organizational aspects of the co-design process were moved to the Methods section, while the Results section was revised to focus more explicitly on findings generated through the participatory process. In the results we also added a table which summarize the changes.

**Reviewer #4**

**Comment:** This study introduces an innovative, co-designed aquatic reactive balance training protocol (Aqua ReBal) that directly addresses major barriers in traditional fall-prevention programs. Exceptional Methodological Rigor: The integration of the GRIPP2 framework guarantees a high standard of transparency and active stakeholder engagement throughout the design process. High Practical Utility: The insights generated from older adult partners offer actionable solutions that significantly enhance participant safety, comfort, and long-term adherence.

**Response:** We sincerely thank the reviewer for the positive and encouraging feedback regarding the novelty, methodological rigor, and practical relevance of the study. We greatly appreciate these comments.

**Reviewer #5**

**Comment:** 1. Lack of rigorous qualitative methodology
The manuscript describes feedback collection through meetings, surveys, and observations, but it does not clearly describe:
• how qualitative data were analyzed,
• whether thematic analysis/content analysis/framework analysis was used,
• who coded the data,
• whether coding was inductive or deductive,
• how trustworthiness/rigor was ensured.
Currently, the Results section largely summarizes discussions narratively rather than presenting a formal qualitative analysis.
Recommendation
The authors should:
• explicitly describe the qualitative analytical approach,
• explain coding procedures,
• clarify how themes/categories were generated,
• include strategies for credibility and rigor (e.g., triangulation, reflexivity, audit trail, member checking).
Without this, the study lacks methodological transparency expected for qualitative/co-design research.

**Response:** Thank you for this important comment. We agree that the qualitative analytical process required clearer description. In response, we expanded the Methods section to clarify that data gathered through participatory sessions, surveys, observations, and field notes were reviewed using an inductive qualitative content analysis approach to support iterative intervention development.

**Comment:** Only three older adult partners participated, all of whom were women and relatively active.
This substantially limits:
• transferability,
• diversity of perspectives,
• applicability to frailer populations,
• gender representation.
While the limitation is acknowledged, the manuscript tends to overstate broader applicability.
Recommendation
The discussion and conclusions should be more cautious regarding generalizability and implementation potential.

**Response:** We agree with the reviewer and revised the manuscript to present conclusions more cautiously. Additional limitations regarding gender representation, transferability, and applicability to frailer populations were added.

**Comment:** The paper describes the co-design process but does not adequately describe the final intervention itself.
Missing details include:
• session duration,
• frequency,
• total intervention dosage,
• perturbation intensity progression,
• therapist-to-participant ratio,
• safety monitoring procedures,
• exact perturbation methods,
• training goals.
For replication and implementation, readers need a clearer description of the finalized protocol.
Recommendation
Consider adding:
• a supplementary table outlining the finalized AquaReBal protocol,
• exercise progression principles,
• safety criteria and stopping rules.

**Response:** Thank you for this valuable suggestion. We agree that clear reporting of the finalized AquaReBal protocol is important for reproducibility and implementation. To address this comment, we added a citation to our recently accepted protocol paper, which provides detailed information regarding the AquaReBal intervention structure, session frequency and duration, exercise progression, perturbation delivery, therapist supervision, and safety procedures:

Ogonowska-Slodownik A, Marinho-Buzelli A, Danells C, Musselman K, Bonnyman A, Alavinia M, Mansfield A. *Feasibility of aquatic reactive balance training (AquaReBal) for older adults: protocol for a single-arm pre-post study*. Gerontology 2026 Mar 4:1-9. doi: 10.1159/000550917.

We also added a brief statement in the manuscript (methods, study design) directing readers to this publication for the full intervention protocol details.

**Comment:** Although participatory action research and co-design are discussed, the manuscript remains largely descriptive and does not critically engage with:
• co-design theory,
• power-sharing dynamics,
• participatory rehabilitation frameworks,
• challenges of stakeholder integration.
The Discussion would benefit from deeper theoretical interpretation.
Recommendation
Expand discussion regarding:
• how older adults influenced decision-making,
• whether any disagreements occurred,
• how consensus was achieved,
• lessons learned about participatory rehabilitation design.

**Response:** Thank you for this thoughtful comment. We agree that the Discussion section required stronger engagement with co-design and participatory rehabilitation concepts beyond the descriptive presentation of the process itself. In response, we expanded the Discussion to more explicitly address the role of older adult partners in shaping intervention decisions, the collaborative and iterative nature of decision-making, and the challenges associated with balancing participant preferences, safety considerations, and feasibility constraints during intervention development.

**Comment:** There is a discrepancy between the submission metadata and manuscript text.
The submission form states:
“All relevant data are within the manuscript and its Supporting Information files.”
However, the manuscript states:
“The data that support the findings of this study are available from the corresponding author upon reasonable request.”
PLOS ONE requires consistency and generally discourages “available on request” statements unless justified.
Recommendation
Revise the data availability statement to comply fully with PLOS ONE policy.

**Response:** Thank you. The Data Availability Statement was revised to ensure consistency with PLOS ONE requirements and submission materials.

**Comment:** Language and grammar issues
Several sentences require proofreading.
Examples:
• “to to shape the program”
• “water environment” should often be “aquatic environment”
• inconsistent use of “partners,” “participants,” and “older adults”
A professional language edit is recommended.

**Response:** The manuscript underwent extensive proofreading and language editing to improve grammar, clarity, and consistency of terminology.

**Comment:** Figures need improvement
Some figures appear visually low-resolution and difficult to interpret:
• Figure 3
• Figure 4
The Miro/Freeform screenshots look informal for journal publication.
Recommendation
Convert figures into professionally formatted thematic diagrams/tables.

**Response:** Thank you for this comment. We acknowledge that Figures 3 and 4 differ from traditional publication-style figures. However, we intentionally retained the Miro and Freeform visual outputs because they represent authentic artifacts generated during the participatory co-design process and illustrate how ideas, barriers, and recommendations emerged collaboratively during the sessions. We believe that preserving these visual materials supports transparency of the co-design methodology and helps readers better understand the iterative and participatory nature of the intervention development process

**Comment:** Limited demographic reporting
The manuscript provides minimal participant characteristics.
Recommendation
Include:
• age range,
• ethnicity,
• mobility status,
• assistive device use,
• fall history,
• aquatic confidence/swimming ability.

**Response:** Thank you for this helpful suggestion. We expanded the participant description in the Methods section to provide additional contextual information regarding the older adult partners involved in the co-design process, including age, fall history, prior experience with aquatic exercise and reactive balance training, and physical activity background.

**Comment:** Given the participatory nature of the study, the manuscript should discuss:
• researchers’ positionality,
• prior relationships with participants,
• potential influence on data interpretation.

**Response:** Thank you for this important comment. We agree that additional reflection regarding researcher positionality and the participatory nature of the study would strengthen the manuscript. In response, we added a reflexivity statement describing the multidisciplinary background of the research team, including expertise in aquatic therapy, reactive balance training, rehabilitation, and patient-oriented research.

**Comment:** Need clearer distinction between feasibility and intervention efficacy
At times, the manuscript implies therapeutic benefit despite no outcome evaluation.
Recommendation
Avoid efficacy-oriented language unless formally tested.
For example:
• “demonstrating potential for broader implementation”
may overstate findings.

**Response:** We agree and revised the manuscript to avoid efficacy-oriented language. Statements implying therapeutic effectiveness or implementation readiness were softened accordingly.

**Section specific comments**

Section-Specific Comments
Abstract
The abstract is clear overall but should mention:
• the qualitative/co-design analytical approach,
• the small sample size as context.

**Response:** Thank you for that comment. It was added in the abstract.

Introduction
Strong rationale overall. However:
• the distinction between aquatic therapy and aquatic reactive balance training should be more explicitly defined,
• stronger mechanistic rationale for why aquatic perturbation training may improve reactive balance would strengthen the paper.

**Response:** Thank you for this helpful comment. We revised the Introduction to more clearly distinguish general aquatic therapy from aquatic reactive balance training (AquaReBal).

Methods
This section requires the most improvement.
Please clarify:
• recruitment strategy,
• inclusion/exclusion criteria,
• qualitative analysis procedures,
• survey questions/scales,
• documentation methods,
• decision-making process for incorporating feedback.

**Response:** All those elements wee addressed in the previous responses.

Results
The Results section currently mixes:
• descriptive reporting,
• interpretation,
• participant quotes.
Consider reorganizing into themes/subthemes.
A thematic table summarizing:
• barriers,
• facilitators,
• design recommendations,
would substantially improve readability.

**Response:** We added the table summarizing the results at the end of the section.

Discussion
The Discussion is generally appropriate but should:
• critically discuss limitations of participatory co-design,
• better integrate qualitative methodology literature,
• avoid overclaiming intervention readiness.

**Response:** All those elements wee addressed in the previous responses.
