# Supplementary material for: Using a co-design approach to develop aquatic reactive balance training for fall prevention
Source: PLoS One. 2026 Jul 15;21(7):e0345435. doi: 10.1371/journal.pone.0345435 (PMC13372110; doi:10.1371/journal.pone.0345435)
Supplement: Peer review history

# Peer review: Decision Letter 0

**DOI:** 10.1371/journal.pone.0345435.r001

**Authors:** Renato Melo

17 May 2026

PONE-D-26-11038

Using a co-design approach to develop aquatic reactive balance training for fall prevention

PLOS One

Dear Dr. Chan Carusone,

Thank you for submitting your manuscript to PLOS ONE. After careful consideration, we feel that it has merit but does not fully meet PLOS ONE’s publication criteria as it currently stands. Therefore, we invite you to submit a revised version of the manuscript that addresses the points raised during the review process.

Please submit your revised manuscript by Jul 01 2026 11:59PM. If you will need more time than this to complete your revisions, please reply to this message or contact the journal office at plosone@plos.org. When you're ready to submit your revision, log on to https://www.editorialmanager.com/pone/ and select the 'Submissions Needing Revision' folder to locate your manuscript file.

Please include the following items when submitting your revised manuscript:

- A letter that responds to each point raised by the academic editor and reviewer(s). You should upload this letter as a separate file labeled 'Response to Reviewers'.
- A marked-up copy of your manuscript that highlights changes made to the original version. You should upload this as a separate file labeled 'Revised Manuscript with Track Changes'.
- An unmarked version of your revised paper without tracked changes. You should upload this as a separate file labeled 'Manuscript'.

If you would like to make changes to your financial disclosure, please include your updated statement in your cover letter. Guidelines for resubmitting your figure files are available below the reviewer comments at the end of this letter.

If applicable, we recommend that you deposit your laboratory protocols in protocols.io to enhance the reproducibility of your results. Protocols.io assigns your protocol its own identifier (DOI) so that it can be cited independently in the future. For instructions see: https://journals.plos.org/plosone/s/submission-guidelines#loc-laboratory-protocols. Additionally, PLOS ONE offers an option for publishing peer-reviewed Lab Protocol articles, which describe protocols hosted on protocols.io. Read more information on sharing protocols at https://plos.org/protocols?utm_medium=editorial-email&utm_source=authorletters&utm_campaign=protocols.

As the corresponding author, your ORCID iD is verified in the submission system and will appear in the published article. PLOS supports the use of ORCID, and we encourage all coauthors to register for an ORCID iD and use it as well. Please encourage your coauthors to verify their ORCID iD within the submission system before final acceptance, as unverified ORCID iDs will not appear in the published article. *Only*  the individual author can complete the verification step; PLOS staff *cannot*  verify ORCID iDs on behalf of authors.

We look forward to receiving your revised manuscript.

Kind regards,

Renato S. Melo, PhD

Academic Editor

PLOS One

Journal Requirements:

When submitting your revision, we need you to address these additional requirements.

1. Please ensure that your manuscript meets PLOS ONE's style requirements, including those for file naming. The PLOS ONE style templates can be found at

https://journals.plos.org/plosone/s/file?id=wjVg/PLOSOne_formatting_sample_main_body.pdf and

https://journals.plos.org/plosone/s/file?id=ba62/PLOSOne_formatting_sample_title_authors_affiliations.pdf

2. PLOS requires an ORCID iD for the corresponding author in Editorial Manager on papers submitted after December 6th, 2016. Please ensure that you have an ORCID iD and that it is validated in Editorial Manager. To do this, go to ‘Update my Information’ (in the upper left-hand corner of the main menu), and click on the Fetch/Validate link next to the ORCID field. This will take you to the ORCID site and allow you to create a new iD or authenticate a pre-existing iD in Editorial Manager.

3. We note that the grant information you provided in the ‘Funding Information’ and ‘Financial Disclosure’ sections do not match.

When you resubmit, please ensure that you provide the correct grant numbers for the awards you received for your study in the ‘Funding Information’ section

4. Thank you for stating the following financial disclosure:

“Polish National Agency for Academic Exchange funded the scholarship for Anna Ogonowska-Slodownik (Bekker Program).”

Please state what role the funders took in the study.  If the funders had no role, please state: "The funders had no role in study design, data collection and analysis, decision to publish, or preparation of the manuscript."

If this statement is not correct you must amend it as needed.

Please include this amended Role of Funder statement in your cover letter; we will change the online submission form on your behalf.

5. Your ethics statement should only appear in the Methods section of your manuscript. If your ethics statement is written in any section besides the Methods, please delete it from any other section.

6. We note that your Data Availability Statement is currently as follows: “All relevant data are within the manuscript and its Supporting Information files.”

Please confirm at this time whether or not your submission contains all raw data required to replicate the results of your study. Authors must share the “minimal data set” for their submission. PLOS defines the minimal data set to consist of the data required to replicate all study findings reported in the article, as well as related metadata and methods (https://journals.plos.org/plosone/s/data-availability#loc-minimal-data-set-definition).

For example, authors should submit the following data:

- The values behind the means, standard deviations and other measures reported;

- The values used to build graphs;

- The points extracted from images for analysis.

Authors do not need to submit their entire data set if only a portion of the data was used in the reported study.

If your submission does not contain these data, please either upload them as Supporting Information files or deposit them to a stable, public repository and provide us with the relevant URLs, DOIs, or accession numbers. For a list of recommended repositories, please see https://journals.plos.org/plosone/s/recommended-repositories.

If there are ethical or legal restrictions on sharing a de-identified data set, please explain them in detail (e.g., data contain potentially sensitive information, data are owned by a third-party organization, etc.) and who has imposed them (e.g., an ethics committee). Please also provide contact information for a data access committee, ethics committee, or other institutional body to which data requests may be sent. If data are owned by a third party, please indicate how others may request data access.

7. If the reviewer comments include a recommendation to cite specific previously published works, please review and evaluate these publications to determine whether they are relevant and should be cited. There is no requirement to cite these works unless the editor has indicated otherwise.

Additional Editor Comments (if provided):

Reviewers' comments:

Reviewer's Responses to Questions

**Comments to the Author**

1. Is the manuscript technically sound, and do the data support the conclusions?

The manuscript must describe a technically sound piece of scientific research with data that supports the conclusions. Experiments must have been conducted rigorously, with appropriate controls, replication, and sample sizes. The conclusions must be drawn appropriately based on the data presented.

Reviewer #1: Yes

Reviewer #2: Yes

Reviewer #3: Yes

Reviewer #4: Yes

Reviewer #5: No

**********

2. Has the statistical analysis been performed appropriately and rigorously?

Reviewer #1: I Don't Know

Reviewer #2: N/A

Reviewer #3: N/A

Reviewer #4: Yes

Reviewer #5: I Don't Know

**********

3. Have the authors made all data underlying the findings in their manuscript fully available?

The PLOS Data policy requires authors to make all data underlying the findings described in their manuscript fully available without restriction, with rare exception (please refer to the Data Availability Statement in the manuscript PDF file). The data should be provided as part of the manuscript or its supporting information, or deposited to a public repository. For example, in addition to summary statistics, the data points behind means, medians and variance measures should be available. If there are restrictions on publicly sharing data—e.g. participant privacy or use of data from a third party—those must be specified.

Reviewer #1: Yes

Reviewer #2: Yes

Reviewer #3: No

Reviewer #4: Yes

Reviewer #5: No

**********

4. Is the manuscript presented in an intelligible fashion and written in standard English?

PLOS ONE does not copyedit accepted manuscripts, so the language in submitted articles must be clear, correct, and unambiguous. Any typographical or grammatical errors should be corrected at revision, so please note any specific errors here.

Reviewer #1: Yes

Reviewer #2: Yes

Reviewer #3: Yes

Reviewer #4: Yes

Reviewer #5: Yes

**********

5. Review Comments to the Author

Please use the space provided to explain your answers to the questions above. You may also include additional comments for the author, including concerns about dual publication, research ethics, or publication ethics. (Please upload your review as an attachment if it exceeds 20,000 characters)

Reviewer #1: This article explores the using of co-design approach to develop aquatic reactive balance training for fall prevention, which holds certain research value. However, the following issues still exist.

1.Among the research participants, there were only 3 elderly individuals, all of whom were female and thus not representative.

2.The key information such as the training structure, frequency, duration, action composition, and safety standards of the final solution of AquaReBal is not clearly presented, which is not conducive to replication and promotion. It is suggested to supplement the core details of the protocol.

Reviewer #2: Introduction:

- Introduction is lengthy with repetition of the main idea of the work done. Please modify.

Methods:

- I am not sure why the authors refer to participating older adults as “partners”. It might be a cultural thing, but it is confusing. Please modify to participants.

- Authors need to declare how their recruitment process went. How did they choose their older adult participants? What were their inclusion and exclusion criteria? Every detail matters.

- Authors did good preparatory steps with consultation of experts related to RBT and aquatic therapy to make an evidence-based intervention. And while the idea of co-designing the program with the recipients in mind is indeed promising. Including very few older adult participants (only 3 women) made the results reflect a mere personal opinion rather than becoming reflective of a group of older adults and limits the value of the work done. Including more older adults and analyzing their sociodemographic and functional levels or adding baseline and post-intervention functional levels of the participants could have added a greater value to this work

Results:

- Authors start the results section with a section related to how information gathering process proceeded. Any information related to the process of the design itself belongs to the methods section. The findings of each step of that process belongs to the results. Please rearrange accordingly.

- Authors should code their participants with numbers and cite that code with their quoted opinion.

- Authors mentioned using Balance Intensity Scale to measure perceived intensity in the results section. Please add any used measurement tool to the methods section as well.

Reviewer #3: The topic addressed in this manuscript is timely, clinically relevant, and aligns with current priorities in rehabilitation and fall prevention, particularly in ageing populations. The focus on aquatic-based balance training and the integration of participatory approaches are valuable and have the potential to contribute meaningfully to both clinical practice and intervention development.

Study design (Lines 83–94)

The study design is not sufficiently or explicitly stated. The authors should clearly define the study design in a single, concise sentence early in the section (e.g., “This study employed a qualitative co-design approach grounded in Participatory Action Research”). The current description combines conceptual frameworks without clearly positioning the study within a recognized methodological category.

Methodological clarity and rigor (Lines 116–151)

While the authors provide a detailed narrative of the co-design process (including participatory design sessions, internal team sessions, and practical testing), the methodological description remains incomplete and lacks sufficient clarity and rigor from a research design perspective.

First, although an initial information-gathering phase is mentioned (literature review and stakeholder meetings), this phase is not adequately described. There is no information on:

- how the literature review was conducted,

- how stakeholder meetings were structured,

- how data from this phase informed subsequent steps.

Second, the co-design process itself is presented as a sequence of activities (PDS1, ITS1, practical sessions, PDS2, ITS2), but important methodological details are missing. Specifically:

- The methods used within the participatory sessions are not clearly described (e.g., whether structured discussions, focus groups, workshops, or specific co-design techniques were used).

- The role of participants (e.g., level of decision-making power vs. advisory input) is not explicitly defined.

Third, although sessions were audio-recorded and supported by notes, there is no clear description of how the data were analyzed. It is unclear:

- whether a qualitative analysis was conducted,

- which analytic approach was used (e.g., thematic analysis, content analysis),

- how insights were translated into concrete design decisions.

Finally, the link between the stated theoretical framework (Participatory Action Research) and the actual procedures is not fully demonstrated. While iterative cycles are implied, the manuscript does not explicitly map the described steps onto PAR phases (e.g., reflection, planning, action).

Relevance and contribution (Lines 74–76; 406–410)

The manuscript clearly emphasizes the value of co-design and participatory approaches; however, the claimed contribution appears to be predominantly methodological rather than clinical. Both in the Introduction and Discussion, the study’s relevance is framed mainly in terms of advancing co-design practices, rather than demonstrating its added value for rehabilitation outcomes.

While these aspects are valuable, they are not sufficiently connected to the clinical significance of the AquaReBal intervention, particularly in terms of expected impact on fall prevention, improvements in balance or functional outcomes, and added value compared to existing rehabilitation approaches. As a result, the manuscript risks being perceived primarily as a process paper, with limited evidence of clinical innovation or advancement.

Results section

The Results section is not clearly delineated from the Methods and currently lacks sufficient reporting of actual findings. A substantial portion of the content describes procedural aspects of the co-design process, which would be more appropriately presented in the Methods section.

In contrast, the results of the co-design process itself are underdeveloped and insufficiently reported. There is limited detail on the specific suggestions made by participants, the nature of the feedback is not clearly categorized, and the concrete changes made to the intervention based on participant input are not systematically presented.

This limits the reader’s ability to understand what was learned from participants, how their contributions shaped the intervention, and the added value of the participatory approach.

Reviewer #4: Significant Clinical Contribution: This study introduces an innovative, co-designed aquatic reactive balance training protocol (Aqua ReBal) that directly addresses major barriers in traditional fall-prevention programs. Exceptional Methodological Rigor: The integration of the GRIPP2 framework guarantees a high standard of transparency and active stakeholder engagement throughout the design process. High Practical Utility: The insights generated from older adult partners offer actionable solutions that significantly enhance participant safety, comfort, and long-term adherence.

Reviewer #5: Using a co-design approach to develop aquatic reactive balance training for fall prevention

Overall Recommendation

Major Revision

This manuscript presents a timely and relevant study describing the co-design of an aquatic reactive balance training (AquaReBal) intervention for older adults. The topic is novel and clinically meaningful, particularly given the increasing interest in participatory rehabilitation research and fall prevention strategies for older adults. The integration of patient/public involvement (PPI) and aquatic therapy concepts is a notable strength.

However, the manuscript currently reads more as a descriptive project report than a rigorously structured scientific study. Several methodological, reporting, and analytical aspects require clarification and strengthening before the work is suitable for publication in PLOS ONE.

General Comments

Strengths

1. Novelty and relevance

o The concept of aquatic reactive balance training is innovative and clinically relevant for older adults with fear of falling or intolerance to land-based perturbation training.

2. Strong patient/public involvement

o The manuscript appropriately integrates older adult partners throughout the intervention development process.

o Use of the GRIPP2 framework is commendable.

3. Clear participatory workflow

o The iterative co-design process is well organized and visually represented in Figure 1.

4. Practical rehabilitation relevance

o The paper provides useful implementation insights for clinicians developing aquatic rehabilitation programs.

Major Concerns

1. Lack of rigorous qualitative methodology

The manuscript describes feedback collection through meetings, surveys, and observations, but it does not clearly describe:

• how qualitative data were analyzed,

• whether thematic analysis/content analysis/framework analysis was used,

• who coded the data,

• whether coding was inductive or deductive,

• how trustworthiness/rigor was ensured.

Currently, the Results section largely summarizes discussions narratively rather than presenting a formal qualitative analysis.

Recommendation

The authors should:

• explicitly describe the qualitative analytical approach,

• explain coding procedures,

• clarify how themes/categories were generated,

• include strategies for credibility and rigor (e.g., triangulation, reflexivity, audit trail, member checking).

Without this, the study lacks methodological transparency expected for qualitative/co-design research.

2. Sample size and representativeness are very limited

Only three older adult partners participated, all of whom were women and relatively active.

This substantially limits:

• transferability,

• diversity of perspectives,

• applicability to frailer populations,

• gender representation.

While the limitation is acknowledged, the manuscript tends to overstate broader applicability.

Recommendation

The discussion and conclusions should be more cautious regarding generalizability and implementation potential.

3. Insufficient detail regarding the final AquaReBal protocol

The paper describes the co-design process but does not adequately describe the final intervention itself.

Missing details include:

• session duration,

• frequency,

• total intervention dosage,

• perturbation intensity progression,

• therapist-to-participant ratio,

• safety monitoring procedures,

• exact perturbation methods,

• training goals.

For replication and implementation, readers need a clearer description of the finalized protocol.

Recommendation

Consider adding:

• a supplementary table outlining the finalized AquaReBal protocol,

• exercise progression principles,

• safety criteria and stopping rules.

4. Limited integration of co-design theory

Although participatory action research and co-design are discussed, the manuscript remains largely descriptive and does not critically engage with:

• co-design theory,

• power-sharing dynamics,

• participatory rehabilitation frameworks,

• challenges of stakeholder integration.

The Discussion would benefit from deeper theoretical interpretation.

Recommendation

Expand discussion regarding:

• how older adults influenced decision-making,

• whether any disagreements occurred,

• how consensus was achieved,

• lessons learned about participatory rehabilitation design.

5. Data availability statement inconsistency

There is a discrepancy between the submission metadata and manuscript text.

The submission form states:

“All relevant data are within the manuscript and its Supporting Information files.”

However, the manuscript states:

“The data that support the findings of this study are available from the corresponding author upon reasonable request.”

PLOS ONE requires consistency and generally discourages “available on request” statements unless justified.

Recommendation

Revise the data availability statement to comply fully with PLOS ONE policy.

Minor Concerns

1. Language and grammar issues

Several sentences require proofreading.

Examples:

• “to to shape the program”

• “water environment” should often be “aquatic environment”

• inconsistent use of “partners,” “participants,” and “older adults”

A professional language edit is recommended.

2. Figures need improvement

Some figures appear visually low-resolution and difficult to interpret:

• Figure 3

• Figure 4

The Miro/Freeform screenshots look informal for journal publication.

Recommendation

Convert figures into professionally formatted thematic diagrams/tables.

3. Limited demographic reporting

The manuscript provides minimal participant characteristics.

Recommendation

Include:

• age range,

• ethnicity,

• mobility status,

• assistive device use,

• fall history,

• aquatic confidence/swimming ability.

4. Reflexivity statement missing

Given the participatory nature of the study, the manuscript should discuss:

• researchers’ positionality,

• prior relationships with participants,

• potential influence on data interpretation.

5. Need clearer distinction between feasibility and intervention efficacy

At times, the manuscript implies therapeutic benefit despite no outcome evaluation.

Recommendation

Avoid efficacy-oriented language unless formally tested.

For example:

• “demonstrating potential for broader implementation”

may overstate findings.

Section-Specific Comments

Abstract

The abstract is clear overall but should mention:

• the qualitative/co-design analytical approach,

• the small sample size as context.

Introduction

Strong rationale overall. However:

• the distinction between aquatic therapy and aquatic reactive balance training should be more explicitly defined,

• stronger mechanistic rationale for why aquatic perturbation training may improve reactive balance would strengthen the paper.

Methods

This section requires the most improvement.

Please clarify:

• recruitment strategy,

• inclusion/exclusion criteria,

• qualitative analysis procedures,

• survey questions/scales,

• documentation methods,

• decision-making process for incorporating feedback.

Results

The Results section currently mixes:

• descriptive reporting,

• interpretation,

• participant quotes.

Consider reorganizing into themes/subthemes.

A thematic table summarizing:

• barriers,

• facilitators,

• design recommendations,

would substantially improve readability.

Discussion

The Discussion is generally appropriate but should:

• critically discuss limitations of participatory co-design,

• better integrate qualitative methodology literature,

• avoid overclaiming intervention readiness.

**********

6. PLOS authors have the option to publish the peer review history of their article (what does this mean?). If published, this will include your full peer review and any attached files.

If you choose “no”, your identity will remain anonymous but your review may still be made public.

**Do you want your identity to be public for this peer review?**  For information about this choice, including consent withdrawal, please see our Privacy Policy.

Reviewer #1: No

Reviewer #2: No

Reviewer #3: No

Reviewer #4: No

Reviewer #5: No

**********

[NOTE: If reviewer comments were submitted as an attachment file, they will be attached to this email and accessible via the submission site. Please log into your account, locate the manuscript record, and check for the action link "View Attachments". If this link does not appear, there are no attachment files.]

To ensure your figures meet our technical requirements, please review our figure guidelines: https://journals.plos.org/plosone/s/figures

You may also use PLOS’s free figure tool, NAAS, to help you prepare publication quality figures: https://journals.plos.org/plosone/s/figures#loc-tools-for-figure-preparation.

NAAS will assess whether your figures meet our technical requirements by comparing each figure against our figure specifications.

Attachment Submitted filename: PONE-D-26-11038.docx

# Author comment: Author response to Decision Letter 1

**DOI:** 10.1371/journal.pone.0345435.r002

10 Jun 2026

Response to Reviewers’ Comments

“Using a co-design approach to develop aquatic reactive balance training for fall prevention”

Thank you for the review of our manuscript. We sincerely appreciate the time, expertise, and constructive feedback provided by the Editor and Reviewers. We carefully considered all comments and revised the manuscript accordingly. We believe the revisions have substantially strengthened the methodological clarity, reporting transparency, and overall quality of the manuscript. All changes made in the revised manuscript are marked as tracked changes. Below, we provide a detailed point-by-point response to each comment

Reviewer #1

Comment: Among the research participants, there were only 3 elderly individuals, all of whom were female and thus not representative.

Response: We thank the reviewer for this important observation. We would like to clarify that the older adults involved in this study were engaged as partners in the co-design process rather than as research participants in an intervention study. Their role was to collaboratively contribute lived experience perspectives to the development and refinement of the AquaReBal program. Nevertheless, we acknowledge that the small number of older adult partners and the inclusion of only women limit the diversity and transferability of perspectives represented in the co-design process. We therefore revised the Limitations section to more explicitly discuss the limited gender representation, the relatively active profile of the partners, and the potential lack of applicability to older adults with greater frailty, different cultural backgrounds, or differing experiences with aquatic exercise. We also clarified that this study aimed to describe an initial co-design process rather than provide representative perspectives of the broader older adult population.

Comment: The key information such as the training structure, frequency, duration, action composition, and safety standards of the final solution of AquaReBal is not clearly presented.

Response: Thank you for this valuable suggestion. We agree that clear reporting of the finalized AquaReBal protocol is important for reproducibility and implementation. To address this comment, we added a citation to our recently accepted protocol paper, which provides detailed information regarding the AquaReBal intervention structure, session frequency and duration, exercise progression, perturbation delivery, therapist supervision, and safety procedures:

Ogonowska-Slodownik A, Marinho-Buzelli A, Danells C, Musselman K, Bonnyman A, Alavinia M, Mansfield A. Feasibility of aquatic reactive balance training (AquaReBal) for older adults: protocol for a single-arm pre-post study. Gerontology 2026 Mar 4:1-9. doi: 10.1159/000550917.

We also added a brief statement in the manuscript (methods, study design) directing readers to this publication for the full intervention protocol details.

Reviewer #2

Introduction

Comment: Introduction is lengthy with repetition of the main idea.

Response: Thank you for this suggestion. The Introduction was revised to improve clarity and conciseness. Repetitive statements were removed, and the rationale was streamlined while maintaining the key theoretical and clinical context.

Comment: I am not sure why the authors refer to participating older adults as “partners”. It might be a cultural thing, but it is confusing. Please modify to participants.

Response: Thank you for this comment. We understand the concern regarding terminology. However, we intentionally retained the term “partners” throughout the manuscript because it aligns with patient and public involvement (PPI) terminology and reflects the collaborative role older adults played in the co-design process rather than their role as passive study participants. To improve clarity, we added an explanation of this terminology in the methods.

Comment:Authors need to declare how their recruitment process went. How did they choose their older adult participants? What were their inclusion and exclusion criteria? Every detail matters.

Response: Thank you. We expanded the Methods section to clearly describe the recruitment.

Comment: Authors did good preparatory steps with consultation of experts related to RBT and aquatic therapy to make an evidence-based intervention. And while the idea of co-designing the program with the recipients in mind is indeed promising. Including very few older adult participants (only 3 women) made the results reflect a mere personal opinion rather than becoming reflective of a group of older adults and limits the value of the work done. Including more older adults and analyzing their sociodemographic and functional levels or adding baseline and post-intervention functional levels of the participants could have added a greater value to this work.

Response: We thank the reviewer for this important observation. We would like to clarify that the older adults involved in this study were engaged as partners in the co-design process rather than as research participants in an intervention study. Their role was to collaboratively contribute lived experience perspectives to the development and refinement of the AquaReBal program. Nevertheless, we acknowledge that the small number of older adult partners and the inclusion of only women limit the diversity and transferability of perspectives represented in the co-design process. We therefore revised the Limitations section to more explicitly discuss the limited gender representation, the relatively active profile of the partners, and the potential lack of applicability to older adults with greater frailty, different cultural backgrounds, or differing experiences with aquatic exercise. We also clarified that this study aimed to describe an initial co-design process rather than provide representative perspectives of the broader older adult population.

Comment: Authors start the results section with a section related to how information gathering process proceeded. Any information related to the process of the design itself belongs to the methods section. The findings of each step of that process belongs to the results. Please rearrange accordingly.

Response: Thank you for this helpful suggestion. We reorganized the manuscript to improve separation between procedural descriptions and findings. Information regarding how the co-design process was conducted was moved to the Methods section, while findings remain in the Results section.

Comment: Participants should be coded with numbers.

Response: Thank you. Participant quotes are now coded consistently (e.g., Partner 1, Partner 2, Partner 3) throughout the manuscript.

Comment: Authors mentioned using Balance Intensity Scale to measure perceived intensity in the results section. Please add any used measurement tool to the methods section as well.

Response: Thank you for noting this omission. Information regarding the Balance Intensity Scale (BIS), has now been added to the Methods section.

Reviewer #3

Comment: The study design is not sufficiently or explicitly stated. The authors should clearly define the study design in a single, concise sentence early in the section (e.g., “This study employed a qualitative co-design approach grounded in Participatory Action Research”). The current description combines conceptual frameworks without clearly positioning the study within a recognized methodological category.

Response: Thank you for this valuable comment. We revised the opening of the Methods section to clearly state the study design.

Comment: While the authors provide a detailed narrative of the co-design process (including participatory design sessions, internal team sessions, and practical testing), the methodological description remains incomplete and lacks sufficient clarity and rigor from a research design perspective.

First, although an initial information-gathering phase is mentioned (literature review and stakeholder meetings), this phase is not adequately described. There is no information on:

- how the literature review was conducted,

- how stakeholder meetings were structured,

- how data from this phase informed subsequent steps.

Response: Thank you for this important comment. We agree that the initial information-gathering phase required further clarification. We expanded the Methods section to provide additional details

Comment: Second, the co-design process itself is presented as a sequence of activities (PDS1, ITS1, practical sessions, PDS2, ITS2), but important methodological details are missing. Specifically:

- The methods used within the participatory sessions are not clearly described (e.g., whether structured discussions, focus groups, workshops, or specific co-design techniques were used).

- The role of participants (e.g., level of decision-making power vs. advisory input) is not explicitly defined.

Response: Thank you for this important comment. We agree that additional methodological detail regarding the co-design sessions improves the clarity and rigor of the manuscript. We revised the Methods section to more clearly describe the structure and facilitation of the participatory design sessions

Comment: Third, although sessions were audio-recorded and supported by notes, there is no clear description of how the data were analyzed. It is unclear:

- whether a qualitative analysis was conducted,

- which analytic approach was used (e.g., thematic analysis, content analysis),

- how insights were translated into concrete design decisions.

Finally, the link between the stated theoretical framework (Participatory Action Research) and the actual procedures is not fully demonstrated. While iterative cycles are implied, the manuscript does not explicitly map the described steps onto PAR phases (e.g., reflection, planning, action).

Response: Thank you for this important comment. We agree that the manuscript required clearer description of the analytical process and stronger alignment between the Participatory Action Research (PAR) framework and the study procedures. We revised the Methods section.

Comment: The manuscript clearly emphasizes the value of co-design and participatory approaches; however, the claimed contribution appears to be predominantly methodological rather than clinical. Both in the Introduction and Discussion, the study’s relevance is framed mainly in terms of advancing co-design practices, rather than demonstrating its added value for rehabilitation outcomes.

While these aspects are valuable, they are not sufficiently connected to the clinical significance of the AquaReBal intervention, particularly in terms of expected impact on fall prevention, improvements in balance or functional outcomes, and added value compared to existing rehabilitation approaches. As a result, the manuscript risks being perceived primarily as a process paper, with limited evidence of clinical innovation or advancement.

Response: Thank you for this thoughtful comment. We agree that the clinical significance of AquaReBal required stronger emphasis throughout the manuscript. In response, we revised the Introduction and Discussion to better articulate the potential rehabilitation relevance of aquatic reactive balance training,

Comment: In contrast, the results of the co-design process itself are underdeveloped and insufficiently reported. There is limited detail on the specific suggestions made by participants, the nature of the feedback is not clearly categorized, and the concrete changes made to the intervention based on participant input are not systematically presented.

This limits the reader’s ability to understand what was learned from participants, how their contributions shaped the intervention, and the added value of the participatory approach.

Response: Thank you for this important and constructive comment. We agree that the distinction between procedural descriptions and findings required further clarification. In response, we substantially revised and reorganized both the Methods and Results sections. Procedural and organizational aspects of the co-design process were moved to the Methods section, while the Results section was revised to focus more explicitly on findings generated through the participatory process. In the results we also added a table which summarize the changes.

Reviewer #4

Comment: This study introduces an innovative, co-designed aquatic reactive balance training protocol (Aqua ReBal) that directly addresses major barriers in traditional fall-prevention programs. Exceptional Methodological Rigor: The integration of the GRIPP2 framework guarantees a high standard of transparency and active stakeholder engagement throughout the design process. High Practical Utility: The insights generated from older adult partners offer actionable solutions that significantly enhance participant safety, comfort, and long-term adherence.

Response: We sincerely thank the reviewer for the positive and encouraging feedback regarding the novelty, methodological rigor, and practical relevance of the study. We greatly appreciate these comments.

Reviewer #5

Comment: 1. Lack of rigorous qualitative methodology

The manuscript describes feedback collection through meetings, surveys, and observations, but it does not clearly describe:

• how qualitative data were analyzed,

• whether thematic analysis/content analysis/framework analysis was used,

• who coded the data,

• whether coding was inductive or deductive,

• how trustworthiness/rigor was ensured.

Currently, the Results section largely summarizes discussions narratively rather than presenting a formal qualitative analysis.

Recommendation

The authors should:

• explicitly describe the qualitative analytical approach,

• explain coding procedures,

• clarify how themes/categories were generated,

• include strategies for credibility and rigor (e.g., triangulation, reflexivity, audit trail, member checking).

Without this, the study lacks methodological transparency expected for qualitative/co-design research.

Response: Thank you for this important comment. We agree that the qualitative analytical process required clearer description. In response, we expanded the Methods section to clarify that data gathered through participatory sessions, surveys, observations, and field notes were reviewed using an inductive qualitative content analysis approach to support iterative intervention development.

Comment: Only three older adult partners participated, all of whom were women and relatively active.

This substantially limits:

• transferability,

• diversity of perspectives,

• applicability to frailer populations,

• gender representation.

While the limitation is acknowledged, the manuscript tends to overstate broader applicability.

Recommendation

The discussion and conclusions should be more cautious regarding generalizability and implementation potential.

Response: We agree with the reviewer and revised the manuscript to present conclusions more cautiously. Additional limitations regarding gender representation, transferability, and applicability to frailer populations were added.

Comment: The paper describes the co-design process but does not adequately describe the final intervention itself.

Missing details include:

• session duration,

• frequency,

• total intervention dosage,

• perturbation intensity progression,

• therapist-to-participant ratio,

• safety monitoring procedures,

• exact perturbation methods,

• training goals.

For replication and implementation, readers need a clearer description of the finalized protocol.

Recommendation

Consider adding:

• a supplementary table outlining the finalized AquaReBal protocol,

• exercise progression principles,

• safety criteria and stopping rules.

Response: Thank you for this valuable suggestion. We agree that clear reporting of the finalized AquaReBal protocol is important for reproducibility and implementation. To address this comment, we added a citation to our recently accepted protocol paper, which provides detailed information regarding the AquaReBal intervention structure, session frequency and duration, exercise progression, perturbation delivery, therapist supervision, and safety procedures:

Ogonowska-Slodownik A, Marinho-Buzelli A, Danells C, Musselman K, Bonnyman A, Alavi

Attachment Submitted filename: Response to Reviewers.docx

# Peer review: Decision Letter 1

**DOI:** 10.1371/journal.pone.0345435.r003

**Authors:** Renato Melo, Renato Melo

29 Jun 2026

Using a co-design approach to develop aquatic reactive balance training for fall prevention

PONE-D-26-11038R1

Dear Dr. Carusone,

We’re pleased to inform you that your manuscript has been judged scientifically suitable for publication and will be formally accepted for publication once it meets all outstanding technical requirements.

Within one week, you’ll receive an e-mail detailing the required amendments. When these have been addressed, you’ll receive a formal acceptance letter and your manuscript will be scheduled for publication.

An invoice will be generated when your article is formally accepted. Please note, if your institution has a publishing partnership with PLOS and your article meets the relevant criteria, all or part of your publication costs will be covered. Please make sure your user information is up-to-date by logging into Editorial Manager at Editorial Manager® and clicking the ‘Update My Information' link at the top of the page. For questions related to billing, please contact billing support.

If your institution or institutions have a press office, please notify them about your upcoming paper to help maximize its impact. If they’ll be preparing press materials, please inform our press team as soon as possible -- no later than 48 hours after receiving the formal acceptance. Your manuscript will remain under strict press embargo until 2 pm Eastern Time on the date of publication. For more information, please contact onepress@plos.org.

Kind regards,

Renato S. Melo, PhD

Academic Editor

PLOS One

Additional Editor Comments (optional):

Reviewers' comments:

Reviewer's Responses to Questions

**Comments to the Author**

1. If the authors have adequately addressed your comments raised in a previous round of review and you feel that this manuscript is now acceptable for publication, you may indicate that here to bypass the “Comments to the Author” section, enter your conflict of interest statement in the “Confidential to Editor” section, and submit your "Accept" recommendation.

Reviewer #1: All comments have been addressed

**********

2. Is the manuscript technically sound, and do the data support the conclusions?

The manuscript must describe a technically sound piece of scientific research with data that supports the conclusions. Experiments must have been conducted rigorously, with appropriate controls, replication, and sample sizes. The conclusions must be drawn appropriately based on the data presented.

Reviewer #1: Partly

**********

3. Has the statistical analysis been performed appropriately and rigorously?

Reviewer #1: Yes

**********

4. Have the authors made all data underlying the findings in their manuscript fully available?

The PLOS Data policy requires authors to make all data underlying the findings described in their manuscript fully available without restriction, with rare exception (please refer to the Data Availability Statement in the manuscript PDF file). The data should be provided as part of the manuscript or its supporting information, or deposited to a public repository. For example, in addition to summary statistics, the data points behind means, medians and variance measures should be available. If there are restrictions on publicly sharing data—e.g. participant privacy or use of data from a third party—those must be specified.

Reviewer #1: Yes

**********

5. Is the manuscript presented in an intelligible fashion and written in standard English?

PLOS ONE does not copyedit accepted manuscripts, so the language in submitted articles must be clear, correct, and unambiguous. Any typographical or grammatical errors should be corrected at revision, so please note any specific errors here.

Reviewer #1: Yes

**********

6. Review Comments to the Author

Please use the space provided to explain your answers to the questions above. You may also include additional comments for the author, including concerns about dual publication, research ethics, or publication ethics. (Please upload your review as an attachment if it exceeds 20,000 characters)

Reviewer #1: The authors did a lot of work on this study. The revised manuscript has been substantial improvement. Thank you�

**********

7. PLOS authors have the option to publish the peer review history of their article (what does this mean?). If published, this will include your full peer review and any attached files.

If you choose “no”, your identity will remain anonymous but your review may still be made public.

**Do you want your identity to be public for this peer review?**  For information about this choice, including consent withdrawal, please see our Privacy Policy.

Reviewer #1: No

**********

# Editor report: Acceptance letter

**DOI:** 10.1371/journal.pone.0345435.r004

**Authors:** Renato Melo, Renato Melo

PONE-D-26-11038R1

PLOS One

Dear Dr. Chan Carusone,

I'm pleased to inform you that your manuscript has been deemed suitable for publication in PLOS One. Congratulations! Your manuscript is now being handed over to our production team.

At this stage, our production department will prepare your paper for publication. This includes ensuring the following:

* All references, tables, and figures are properly cited

* All relevant supporting information is included in the manuscript submission,

* There are no issues that prevent the paper from being properly typeset

You will receive further instructions from the production team, including instructions on how to review your proof when it is ready. Please keep in mind that we are working through a large volume of accepted articles, so please give us a few days to review your paper and let you know the next and final steps.

Lastly, if your institution or institutions have a press office, please let them know about your upcoming paper now to help maximize its impact. If they'll be preparing press materials, please inform our press team within the next 48 hours. Your manuscript will remain under strict press embargo until 2 pm Eastern Time on the date of publication. For more information, please contact onepress@plos.org.

You will receive an invoice from PLOS for your publication fee after your manuscript has reached the completed accept phase. If you receive an email requesting payment before acceptance or for any other service, this may be a phishing scheme. Learn how to identify phishing emails and protect your accounts at https://explore.plos.org/phishing.

If we can help with anything else, please email us at customercare@plos.org.

Thank you for submitting your work to PLOS ONE and supporting open access.

Kind regards,

PLOS ONE Editorial Office Staff

on behalf of

Dr. Renato S. Melo

Academic Editor

PLOS One
